# Cross-reactive neutralizing human monoclonal antibodies mapping to variable antigenic sites on the norovirus major capsid protein

**DOI:** 10.3389/fimmu.2022.1040836

**Published:** 2022-10-25

**Authors:** Lauren A. Ford-Siltz, Kentaro Tohma, Gabriela S. Alvarado, Joseph A. Kendra, Kelsey A. Pilewski, James E. Crowe, Gabriel I. Parra

**Affiliations:** ^1^ Division of Viral Products, Center for Biologics Evaluation and Research, Food and Drug Administration, Silver Spring, MD, United States; ^2^ Department of Pathology, Microbiology, and Immunology, Vanderbilt University Medical Center, Nashville, TN, United States; ^3^ Department of Pediatrics and Vanderbilt Vaccine Center, Vanderbilt University Medical Center, Nashville, TN, United States

**Keywords:** norovirus, antibodies, neutralization, GII.4, cross-reactive, mapping, gastroenteritis

## Abstract

Human noroviruses are the major viral cause of acute gastroenteritis around the world. Although norovirus symptoms are in most cases mild and self-limited, severe and prolonged symptoms can occur in the elderly and in immunocompromised individuals. Thus, there is a great need for the development of specific therapeutics that can help mitigate infection. In this study, we sought to characterize a panel of human monoclonal antibodies (mAbs; NORO-123, -115, -273A, -263, -315B, and -250B) that showed carbohydrate blocking activity against the current pandemic variant, GII.4 Sydney 2012. All antibodies tested showed potent neutralization against GII.4 Sydney virus in human intestinal enteroid culture. While all mAbs recognized only GII.4 viruses, they exhibited differential binding patterns against a panel of virus-like particles (VLPs) representing major and minor GII.4 variants spanning twenty-five years. Using mutant VLPs, we mapped five of the mAbs to variable antigenic sites A (NORO-123, -263, -315B, and -250B) or C (NORO-115) on the major capsid protein. Those mapping to the antigenic site A showed blocking activity against multiple variants dating back to 1987, with one mAb (NORO-123) showing reactivity to all variants tested. NORO-115, which maps to antigenic site C, showed reactivity against multiple variants due to the low susceptibility for mutations presented by naturally-occurring variants at the proposed binding site. Notably, we show that cross-blocking and neutralizing antibodies can be elicited against variable antigenic sites. These data provide new insights into norovirus immunity and suggest potential for the development of cross-protective vaccines and therapeutics.

## Introduction

Noroviruses are the major cause of acute gastroenteritis in all age-groups. In healthy individuals, norovirus disease symptoms (diarrhea, vomiting, cramps, and abdominal pain) are self-limited to 2-3 days and vary from mild to moderate in severity. In contrast, norovirus symptoms can be prolonged and life-threatening in immunocompromised individuals, the elderly, and malnourished children (1). Notably, immunocompromised individuals can be chronically infected with norovirus for years, causing complications for the clinical management of this susceptible population (1).

Despite the great disease burden, vaccines and specific therapeutics are not yet available for noroviruses. One of the obstacles for the development of therapeutics or preventive vaccines is the extensive genetic and antigenic diversity presented by norovirus strains (2, 3). Over 30 virus genotypes can infect humans, and while predominance of each genotype can vary within different spatiotemporal settings, GII.4 is the predominant genotype infecting humans for over 2-3 decades. The predominance of GII.4 noroviruses has been linked to the chronological emergence of variants. Thus, since the 1980’s, six major GII.4 virus variants have emerged and caused large outbreaks worldwide: Grimsby 1995, Farmington Hills 2002, Hunter 2004, Den Haag 2006b, New Orleans 2009, and Sydney 2012. Other variants also have been reported (Camberwell 1987, Sakai 2003, Osaka 2007, Yerseke 2006a, and Apeldoorn 2007), but the reason for their limited dispersion and incidence in gastroenteritis is not well understood (4). Most of the differences among these variants map to five variable antigenic sites (designated A, C, D, E, and G) located on the outermost region of the viral capsid protein VP1 (5, 6). Antigenic site A consists of residues 294-298, 368, 372, and 373; site C consists of residues 339-341 and 375-378; site D consists of residues 393-397; site E consists of residues 407 and 411-414; and site G consists of residues 352, 355-357, 359, and 364 (7). These antigenic sites were identified using bioinformatics and were experimentally confirmed with multiple monoclonal antibodies (5, 6, 8–13). Most residues from these antigenic sites map on loops and play a minimal role in the structural integrity of the capsid protein, which explains their flexibility to acquire mutations (14). Recent studies that examined a large collection of viruses showed major shifts in the antigenic properties throughout the evolution of the GII.4 variants. These antigenic differences were associated with amino acid changes occurring in synchrony in multiple antigenic sites during the emergence of these variants (5, 15).

A major goal of current GII.4 norovirus research is the identification and development of protective antibodies that target conserved regions of the VP1 protein. VP1 is composed of two structural domains, the shell and the protruding (P) domains (16). The shell is a highly conserved domain that forms the scaffold of the virus particle, while the P domain, which can be further divided into the P1 and P2 subdomains, is highly variable and contains most determinants of virus:host interaction during the early stages of infection. One of those determinants is the binding to histo-blood group antigen (HGBA) carbohydrates, which are molecules that facilitate norovirus infection (17–19). Unfortunately, only antibodies targeting the P domain seem to be involved in protection as measured by carbohydrate blocking assay or neutralization activity in the enteroid cell culture system (20–23). Multiple mouse and human cross-reactive monoclonal antibodies (mAbs) have been reported (24), but only a fraction of them have been shown to have potential neutralizing activity (25–27). One human neutralizing antibody cross-reacts with multiple GII.4 viruses, and this antibody maps to a conserved region of the P domain (25). Moreover, a human antibody that binds to different norovirus genotypes has shown neutralizing activity, providing hope for the development of cross-protective norovirus vaccines (26).

In this study, we mapped the antigen binding site of five human mAbs developed from individuals that presented with symptomatic norovirus gastroenteritis in 2013 (27). Notably, while the majority of these mAbs mapped to highly variable antigenic sites, they reacted with multiple GII.4 variants that emerged over 20 years prior to the infecting strain. These data provide new insights on the immune response during natural infection with human norovirus that could facilitate the development of efficient therapeutic antibodies and cross-protective vaccines.

## Materials and methods

### VP1 sequence analysis

The VP1-encoding sequences of GII.4 viruses were collected from GenBank and aligned with ClustalW as implemented in MEGA7 (28). The structural model of the GII.4 norovirus P domain dimer (Protein Data Bank accession number 4op7) was rendered using UCSF Chimera (29).

### Wild-type and mutant VLPs production

The VLPs of GII.4 viruses (**Table 1**) were produced using Bac-to-Bac Baculovirus Expression System (Invitrogen) as described previously (15). Briefly, the VP1-encoding sequences were synthesized and cloned into pFastBac1 vectors (GenScript) and transformed into MAX Efficiency DH10Bac cells (Invitrogen). The isolated bacmid was transfected into Sf9 cells to produce VP1 proteins that formed VLPs. The produced VLPs were purified using sucrose cushions and cesium chloride gradients, followed by dialysis with 1× PBS (pH 7.4, Gibco). Mutant VLPs were produced by synthesizing the VP1-encoding sequences with antigenic sites transplanted from different viruses or mutated to alanine (13). Similarly, VLPs from other genotypes, including GI.1, GII.1, GII.2, GII.6, GII.12, and GII.17 (**Table 1**), were produced using publicly available VP1-encoding sequences (23). All VLPs were checked for integrity using transmission electron microscopy.

**Table 1:** Information on GII.4 and non-GII.4 viruses and the associated GenBank accession numbers used to produce the VLPs that are referenced in this study.

Genotype or GII.4 variant	Virus	Year	Accession Number
GI.1	8FIIa	1968	JX023285
GII.1	7EK-Hawaii	1971	JX289822
GII.2	HenrytonSP17	1971	MF405169
GII.6	BethesdaD1	2012	KY424341
GII.12	HS210	2010	HQ449728
GII.17	Gaithersburg	2014	KR083017
Sydney (SY) 2012	RockvilleD1	2012	KY424328
New Orleans (NO) 2009	Virginia	2010	KX353958
	Ehime2	2009	AB933752
Apeldoorn (AP) 2007	Iwate4	2008	AB541274
Osaka (OS) 2007	Osaka	2007	AB434770
Den Haag (DH) 2006b	Den Haag89	2006	EF126965
Yerseke (YE) 2006a	Yerseke38	2006	EF126963
Hunter (HT) 2004	Nijmegen083	2004	AB303941
Sakai (SA) 2003	Sakai	2005	AB220922
Farmington Hills (FH) 2002	MD2004-3	2004	DQ658413
Grimsby (GR) 1995	Wild type: Grimsby Swapped mutant: Arizona	1995 1996	AJ004864 AF080556
Camberwell (CA) 1987	MD145-12	1987	AY032605

### Human mAb production

Human mAbs were isolated previously from healthy adults who were naturally infected with a GII.4 Sydney virus in 2013 using an established human B cell hybridoma technique (27). Here, cloned hybridoma cell lines that had been cryopreserved were thawed and placed into culture and expanded gradually from 48-well plates to 12-well plates, T-25, T-75, and eventually to multiple T-225 flasks for each cell line. Following 4 weeks of incubation at 37°C, supernatant from the T-225 flasks was harvested and filtered through a 0.4-µm filter. The supernatant was purified using column chromatography, specifically HiTrap KappaSelect and Lambda FabSelect affinity resins (GE Healthcare Life Sciences).

### mAb-binding ELISA

To profile the binding pattern of human mAbs against the VLPs, ELISAs with wild-type and mutant VLPs were performed as described previously (5). Briefly, 96-well “U” bottom plates were coated with 0.5 μg/mL of VLPs in 1× PBS (pH 7.4, Gibco) overnight at 4°C and blocked for one hour at room temperature with 5% blocking buffer (Bio-Rad). Human mAbs (2 μg/mL each or serial dilutions starting at 2 μg/mL) were plated in duplicate and incubated with VLPs for two hours at room temperature. Plates were washed three times with 1× PBS (pH 7.4) containing 0.1% Tween 20. Binding of mAbs to VLPs was visualized using 1:2,000 anti-human IgG, IgA, IgM conjugated with horseradish peroxidase (HRP) and ABTS 1-Component Microwell Peroxidase Substrate (SeraCare) and was quantified as an optical density at 405 nm (OD 405 nm) using a SPECTROstar Nano plate reader (BMG LABTECH). The 50% effective concentration (EC_50_) values, or concentrations at which the OD reading was 50% of the OD value at antibody saturation per plate, were calculated using GraphPad Prism v9 (California, USA).

### HBGA-blocking assay

Human saliva contains epithelial cells that expresses HBGA carbohydrates. Thus, HBGA-binding and -blocking assays were performed using HBGA carbohydrates derived from human saliva as described previously (23). Human saliva was collected from a healthy adult volunteer, which showed positive signals against anti-Lewis b, Lewis y, Lewis a (Millipore Sigma), H type-1, and H type-2 antibodies (Invitrogen) in ELISA. The use of human saliva was approved by the Institutional Review Board (protocol number CBER IRB 16-069B). The saliva sample was boiled at 100°C for 10 min immediately after the collection and centrifuged at 13,000 rpm for 5 min. Saliva supernatant (1:200 in 1× PBS, pH 7.4) was then coated on 96-well “U” bottom plates overnight at 4 °C and blocked for 1 hour at room temperature with 5% blocking buffer (Bio-Rad). Two-fold serial dilution of human mAbs (50 μg/mL or 12.5 μg/mL) was plated in duplicate and incubated with VLPs at 0.5 μg/mL for 1 hour at 37°C. Plates were washed four times with 1× PBS (pH 7.4) containing 0.1% Tween 20. Pooled sera from guinea pigs immunized with GII.4 VLPs were used as primary detection antibodies against bound VLPs. Binding (blocking of binding) of VLPs on HBGA carbohydrates was visualized using goat anti-guinea pig IgG-HRP and ABTS (SeraCare). The OD values (OD 405 nm) were quantified and normalized with values from negative and positive controls to calculate the EC_50_, or the concentrations at which the OD reading was 50% of the positive control. EC_50_ values were calculated using GraphPad Prism v9 (California, USA).

### Neutralization assay using human intestinal enteroids system

Human jejunal enteroids (J2) were kindly provided by Dr. Mary Estes (Baylor College of Medicine, Texas). A human intestinal enteroid neutralization assay was performed as previously described (23). Briefly, human intestinal enteroids were maintained as three-dimensional cultures in Human IntestiCult Organoid Growth Medium (Components A and B; Stem Cell Technologies). For neutralization experiments, human intestinal enteroids were plated as monolayers on 96-well plates. Monolayers were differentiated for 6 to 7 days until confluent in 1:1 of Component A and CMGF- medium (Advanced Dulbecco Modified Eagle Medium (DMEM/F12) enriched with 1% GlutaMAX, 1% 1M HEPES, and 1% penicillin/streptomycin, Thermo Fisher Scientific). The monolayers were incubated in this differentiation medium + 0.5 mM of glycochenodeoxycholic acid (GCDCA), a glycine-conjugated form of the primary bile acid chenodeoxycholic acid, for 2 to 4 days prior to infection/neutralization. A Sydney 2012 variant virus, GII.4 011617/USA/2017 (GenBank Accession Number: MN782359), was filtered from stool suspensions and was used for all neutralization experiments (23). The use of human stool was approved by the Institutional Review Board (protocol number CBER IRB 16-069B). For neutralization, the virus (with an input titer of approximately 156 genome copies/µL) was incubated with serial dilutions of human mAbs for 1 hour. Two independent experiments were performed. For the first experiment, the initial starting mAbs concentration was 10 μg/ml. The second experiment was performed with either 10 μg/ml or 0.1 μg/ml, depending on the potency of the mAb as determined by the first experiment. Human intestinal enteroids were inoculated with 100 µL of virus or virus-mAb mixes (+ 0.5 mM GCDCA) and incubated at 37°C to allow for virus adsorption. Following washes, the monolayers were overlaid with fresh differentiation medium (+ 0.5 mM GCDCA) and incubated at 37°C for 3 days. At 1 hour post-inoculation (HPI) or 3 days post-infection (DPI), the cells were thawed and frozen three times. Viral RNA was extracted from whole-cell content using the MagMAX-96 Viral RNA Isolation Kit (Applied Biosystem). Quantitative RT-PCR was performed as described previously with a full-length GII.4 RNA transcript as the standard curve (23, 30). The results were analyzed with CFX Maestro software (Bio-Rad) and visualized in GraphPad Prism v9.

## Results

### Human mAbs isolated from individuals infected with GII.4 Sydney variant specifically block and neutralize GII.4 norovirus

A panel of twenty-five IgG and IgA mAbs was isolated from humans naturally infected with a GII.4 Sydney variant. The majority of these mAbs exhibited carbohydrate blockade activity and several neutralized viruses in human intestinal enteroid cultures (27). Based on competition-binding analyses, these antibodies showed three binding patterns on the VP1 protein. We hypothesized here that antibodies from each of the competition groups would map to different antigenic sites (A, C, D, E, or G) on the VP1. Thus, we sought to characterize a subset of P-domain-binding IgG (NORO-123, -115, -263, -315B, and -250B) and IgA (NORO-273A) mAbs that showed different binding profiles (27).

**Figure 1:**
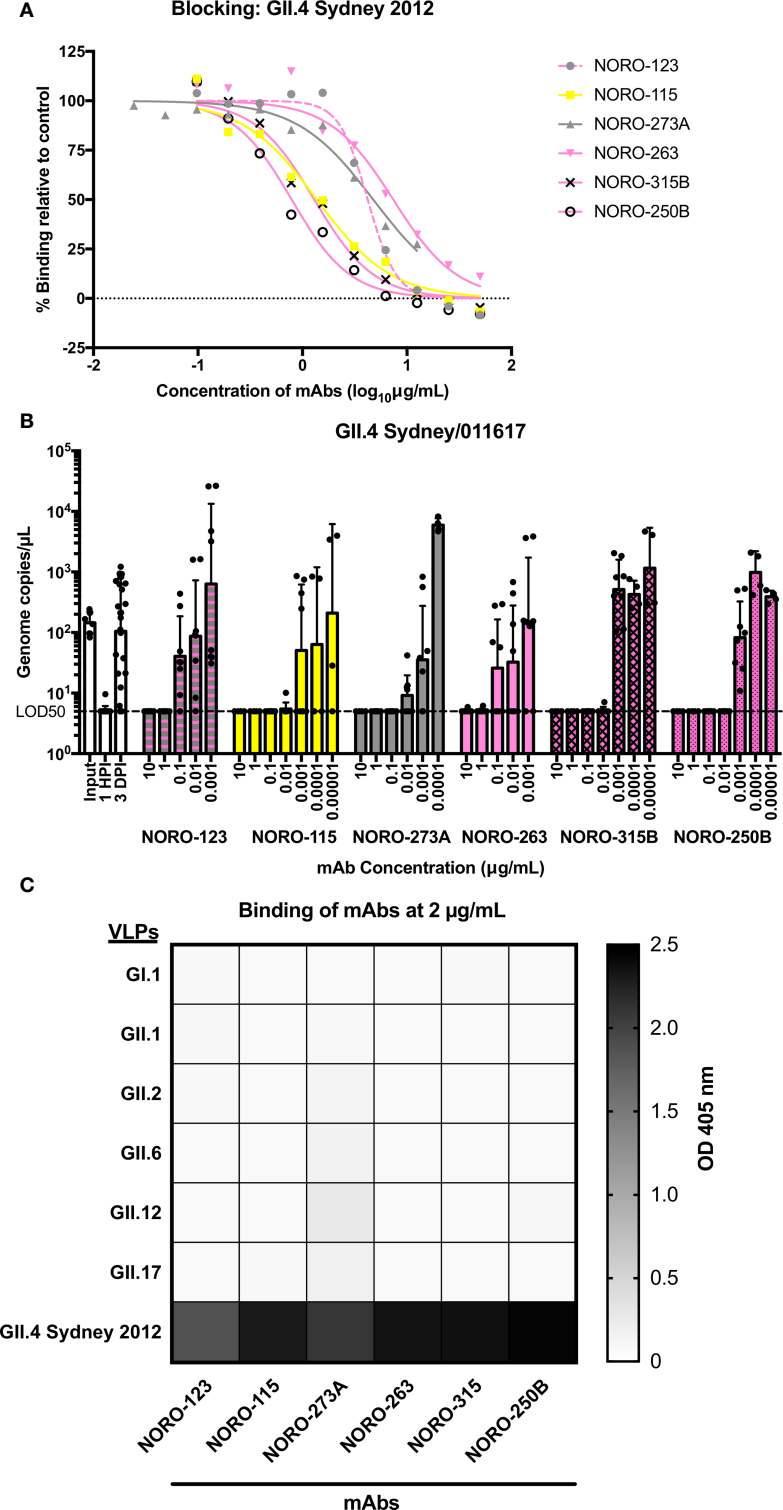
Human mAbs isolated from individuals infected with a GII.4 Sydney 2012 variant exhibit blocking and neutralization against GII.4 Sydney 2012, but not against non-GII.4 genotypes. **(A)** Carbohydrate blocking of human mAbs against a GII.4 Sydney 2012 variant virus (RockvilleD1/2012). Blocking of serial dilutions of mAbs (starting at 50 μg/mL or 12.5 μg/mL) was determined by comparing the signal to that of the positive control, binding of VLPs in the absence of mAbs. **(B)** Neutralization of a GII.4 Sydney 2012 virus (strain 011617) with different concentrations of human mAbs. The dashed line represents 5 genome copies/µL, which is the copy number per µL for which the limit of detection is 50% (LOD_50_) or the “neutralization threshold”, which was defined as the average of genome copies/µL after 1 hour post-inoculation (HPI) such that samples presenting values below the line were unable to replicate and were therefore neutralized. All virus inoculations were performed in the presence of 0.5 mM glycochenodeoxycholic acid (GCDCA), a glycine-conjugated form of the primary bile acid chenodeoxycholic acid. The graph represents the average values of two independent experiments. The error bars represent the geometric standard deviation of the mean. **(C)** Heat map of the binding of mAbs (at 2 μg/mL) to VLPs of selected genotypes. The value in each cell is the average of the optical density at 405 nm (OD 405 nm) in duplicate wells.

All six mAbs blocked the HBGA binding of GII.4 Sydney 2012 virus-like particles (VLPs) based on the RockvilleD1/2012 virus (**Figure 1A**), with half-maximal effective concentration (EC_50_) blockade values ranging from 0.8 to 7.4 μg/mL. To determine the neutralizing capacity of the mAbs, we inoculated monolayers of human intestinal enteroid cells with GII.4/011617, a Sydney 2012 virus detected in 2017, in the presence or absence of different concentrations of mAbs. The GII.4/011617 virus differs from RockvilleD1/2012 by only three amino acid residues on VP1 (S6N, P174S, and N285T), with none of them in known antigenic sites. Like the results from the blockade assay, all six mAbs neutralized the GII.4 Sydney 2012 virus (**Figure 1B**), reinforcing the concept that carbohydrate blocking activity correlates with viral neutralization (13, 23, 31).

Although GII.4 viruses are the most common cause of norovirus outbreaks worldwide, humans are often exposed to viruses from multiple genotypes throughout their lifetimes (32–36). As these mAbs were isolated from immunocompetent adults, the humoral response may have been influenced by previous norovirus infections. A binding assay tested against representative VLPs from prototype (GI.1, GII.1) and prevalent genotypes (GII.2, GII.6, GII.12, and GII.17; **Figure 1C**) showed that all mAbs bound exclusively to GII.4 VLPs.

### Human mAbs present blocking activity against multiple GII.4 variants spanning decades

**Figure 2:**
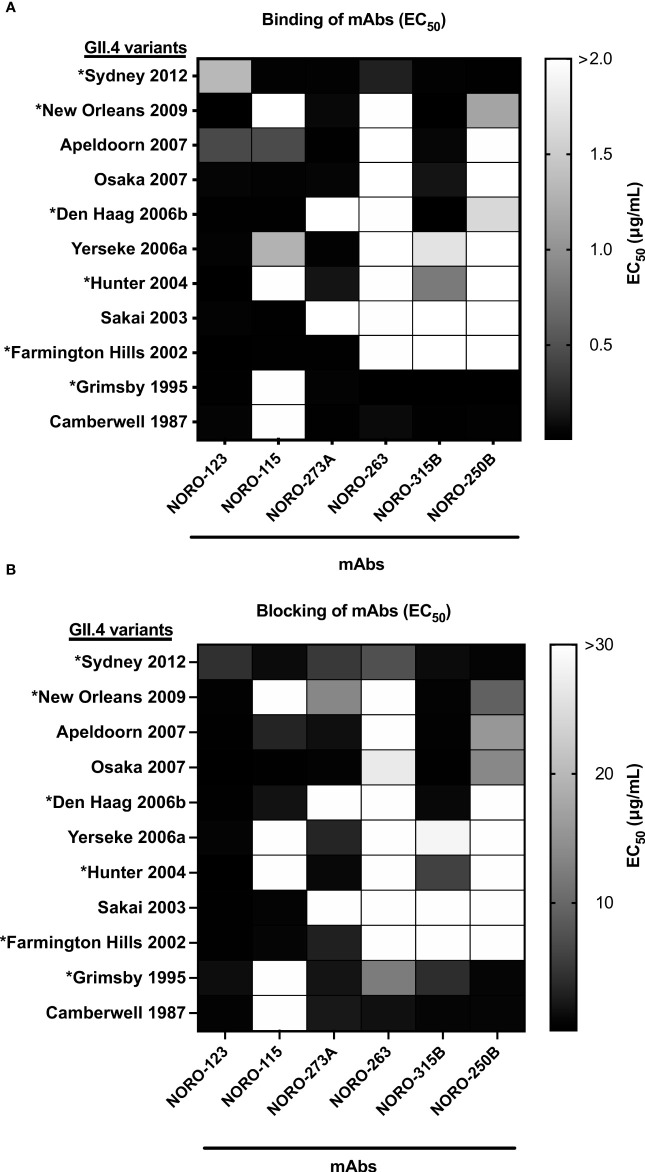
Human mAbs present varying binding and blocking profiles against multiple GII.4 variants spanning over three decades. **(A)** Heat map of the binding of mAbs to VLPs representing major and minor GII.4 variants. Each cell shows the average of the EC_50_ values calculated as the half-maximal effective concentration that results in 50% binding to VLPs in duplicate wells, where 100% binding was set as the maximum average OD 405 nm value calculated from each plate at or near antibody saturation. Major pandemic variants are labeled with an asterisk. **(B)** Heat map of the carbohydrate blocking of human mAbs against VLPs representing major and minor GII.4 variants. Blocking of serial dilutions of mAbs was compared to the signal of the positive control, binding of VLPs in the absence of mAbs. Each cell shows the average of the EC_50_ values calculated as the half-maximal effective concentration that results in 50% blocking of VLPs in duplicate wells. Major pandemic variants are labeled with an asterisk.

Similar to influenza viruses, new variants of GII.4 norovirus emerge periodically to predominate in the population (4). To determine the cross-reactivity within GII.4, we tested reactivity against a panel of GII.4 VLPs representing major (denoted with an asterisk) and minor pandemic variants reported since the 1980s: Camberwell (CA) 1987, Grimsby (GR) 1995 (*), Farmington Hills (FH) 2002 (*), Sakai (SA) 2003, Hunter (HT) 2004 (*), Yerseke (YE) 2006a, Den Haag (DH) 2006b (*), Osaka (OS) 2007, Apeldoorn (AP) 2007, New Orleans (NO) 2009 (*), and Sydney (SY) 2012 (*). We first compared the variant binding profile of NORO-273A IgA and the IgG isotype. The variant binding profiles were identical between the IgG and IgA isotypes (**S1 Figure**). Thus, we used the IgG isotype for consistency with the rest of the experiments. All mAbs exhibited various binding patterns with the panel of VLPs (**Figure 2A**), with NORO-123 showing reactivity with all GII.4 variants tested. Notably, the ability to block HBGA carbohydrate interaction correlated with the binding profiles (**Figure 2B**; **S2 Figure**). Despite being isolated from individuals infected with a GII.4 Sydney variant, all mAbs except NORO-115 blocked GII.4 Camberwell 1987 and GII.4 Grimsby 1995 VLPs. Interestingly, NORO-123 blocked all variants tested, with EC_50_ values ranging from 0.11 to 4.2 μg/ml. NORO-263 and -250B presented similar binding and blocking profiles, and the other antibodies showed unique reactivity patterns against the archival and contemporary variants. The majority of antibodies that presented strong binding (EC50 values <1 μg/ml) against the different variants also presented blocking activity against those variants. NORO-250B presented blocking activity against OS 2007 and AP 2007, but binding to these VLPs required higher concentrations (EC_50_ values of 3.8 and 2.8 μg/ml, respectively; **S3 Figure**). Together, these data suggest that these mAbs are broadly blocking, and most likely neutralizing, against GII.4 variants spanning decades.

### Cross-reactive mAbs map to variable antigenic sites on norovirus VP1

**Figure 3:**
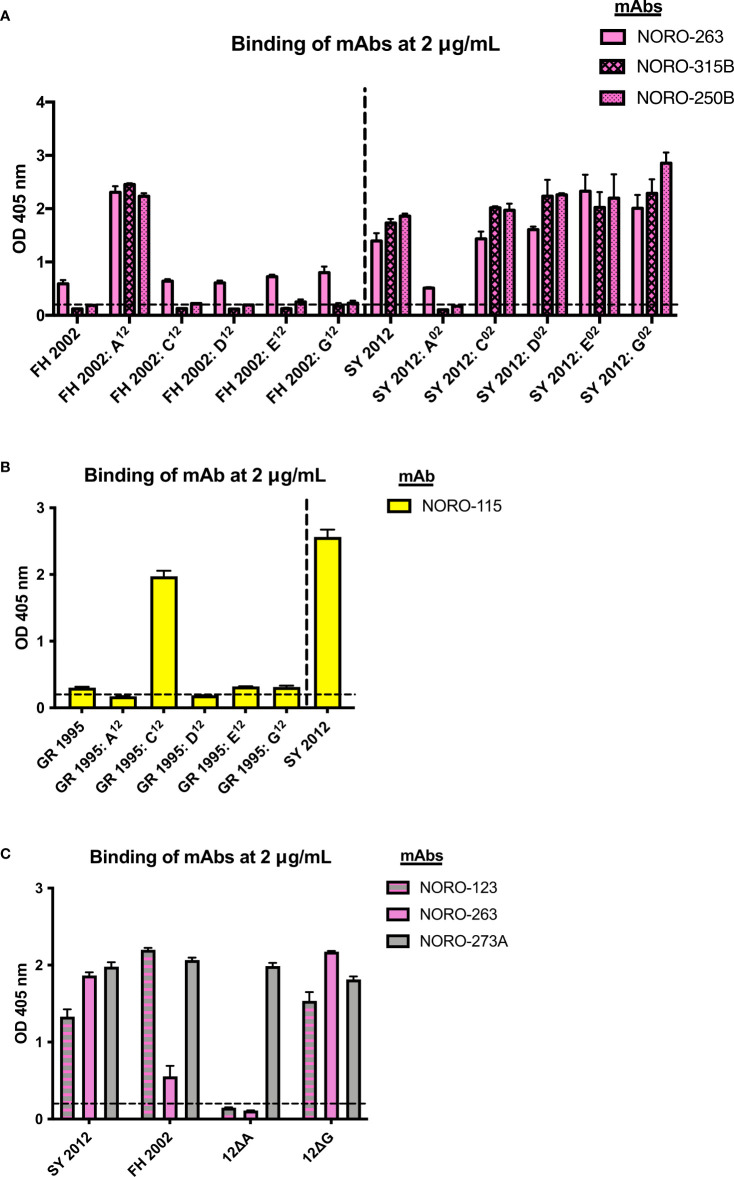
Human mAbs NORO-123, -263, -315B, and -250B bind to antigenic site A and NORO-115 binds to antigenic site C on the major capsid protein. **(A)** Binding of mAbs NORO-263, -315B, and -250B to wild-type (wt) VLPs from Farmington Hills (FH 2002) and Sydney (SY 2012) variants, or to mutant VLPs with swapped antigenic sites A, C, D, E, and G. **(B)** Binding of NORO-115 to wt VLPs from Grimsby (GR 1995) and SY 2012 variants, or to mutant VLPs with antigenic sites A, C, D, E, and G derived from the SY 2012 variant. **(C)** Binding of NORO-123 to SY 2012 VLPs presenting depleted antigenic sites A or G. Values represent the average binding at 2 mg/mL of each mAb. Each bar represents an average of duplicate wells. The dashed line represents the cut-off value (OD 405 nm = 0.2). The error bars represent the standard deviation of the mean.

The GII.4 norovirus variants are mainly characterized by changes on the major antigenic sites (A, C, D, E, and G) of the VP1 protein (5, 15). Thus, these antigenic sites are thought to be major targets of the humoral immune response upon infection. The majority (57%) of a panel of mouse mAbs isolated from mice immunized with GII.4 Sydney variant map to one of these variable antigenic sites, with antigenic sites A and G exhibiting the strongest immunodominance (5, 13). To map the binding sites of the human mAbs, we used a panel of mutant VLPs with swapped residues from each of the antigenic sites (5, 13). For example, the Farmington Hills (FH) 2002: A^12^ VLP consists of the FH backbone with all residues from antigenic site A from the Sydney (SY) 2012 variant, and *vice versa* for SY 2012: A^02^. Because NORO-123, -115, and -273A bind to both Farmington Hills variant (FH 2002) and Sydney variant (SY 2012) VLPs, we could not determine the binding sites for these antibodies using this panel (**S4 Figure**). However, as NORO-263, -315B, and -250B do not bind to FH 2002 VLPs (**Figure 2A**), we showed that swapping the antigenic site A from Farmington Hills 2002 to the Sydney 2012 backbone results in the loss of binding of these three mAbs (**Figure 3A**, SY 2012: A^02^ VLPs). Similarly, addition of the Sydney 2012 antigenic site A to the Farmington Hills 2002 backbone rescues the binding (**Figure 3A**, FH 2002: A^12^ VLPs), confirming that NORO-263, -315B, and -250B all bind to antigenic site A. As NORO-115 does not bind to the GII.4 Grimsby 1995 variant (**Figure 2A**), we performed a binding assay using Grimsby VLPs (GR 1995) with swapped antigenic sites from the Sydney 2012 variant. Interestingly, Grimsby 1995 VLPs with the antigenic site C from the Sydney 2012 variant rescued binding almost to the level seen with SY 2012 VLPs (**Figure 3B**).

As previously described, NORO-123 and -273A bind to both Grimsby 1995 and Farmington Hills 2002 variants (**Figure 2A**); thus, we were unable to determine their binding sites using the swapped mutant VLPs. As an alternative, we performed a binding assay using Sydney 2012 VLPs with all residues on antigenic sites A or G mutated to alanine (12ΔA and 12ΔG, respectively) (13). Although NORO-273A bound to all mutant VLPs, the deletion of antigenic site A surprisingly abrogated the binding of the cross-reactive NORO-123 (**Figure 3C**).

**Figure 4:**
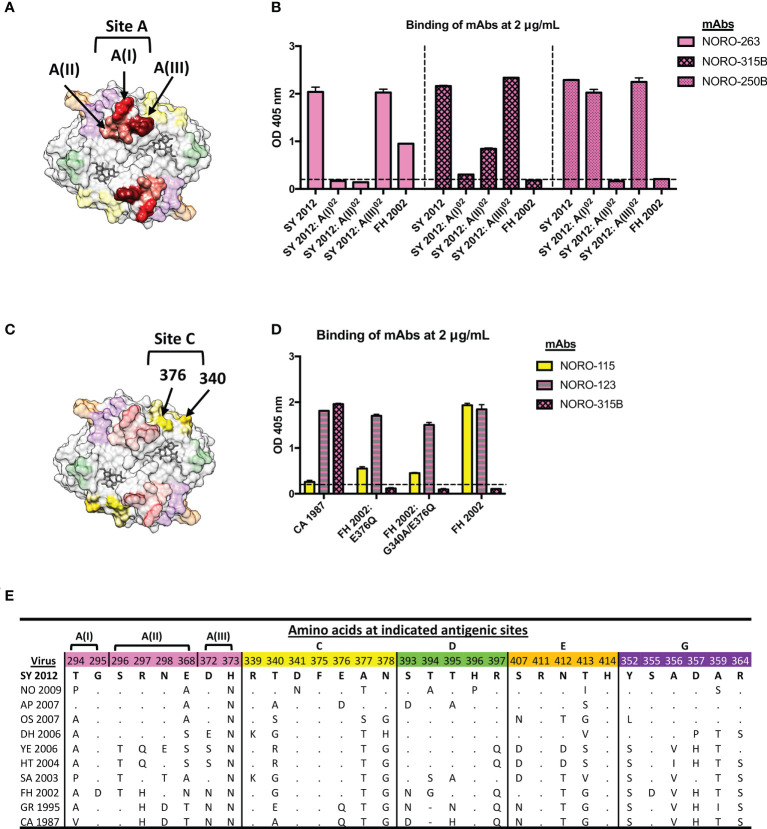
NORO-263, -315B, and -250B bind to motifs A(I) and/or A(II), while residues 376 and 340 contribute to the binding of NORO-115 to antigenic site C. **(A)** Structure of the P domain dimer (top view) with motifs A(I), A(II), and A(III) highlighted in red, pink, and maroon, respectively. The HBGA molecule is represented in dark grey. **(B)** Binding of mAbs NORO-263, -315B, and -250B to wt VLPs from Farmington Hills (FH 2002) and Sydney (SY 2012) variants, or to mutant VLPs with swapped motifs A(I), A(II), or A(III). Values represent the average binding at 2 μg/mL of each mAb. Each bar represents an average of duplicate wells. The dashed line represents the cut-off value (OD 405 nm = 0.2). The error bars represent the standard deviation of the mean. **(C)** Structure of the P domain dimer (top view) with residues 376 and 340 from antigenic site C (yellow) highlighted in gold. An HBGA molecule is represented in dark grey. **(D)** Binding of mAb NORO-115 to wt VLPs from Farmington Hills (FH), Camberwell (CA) variants, or to mutant VLPs presenting the backbone of FH variant with residues exchanged with the CA variant (FH 2002: E376Q and FH 2002: G340A/E376Q). The error bars represent the standard deviation of the mean. **(E)** Sequence alignment of antigenic sites A, C, D, E, and G from selected GII.4 viruses. CA, Camberwell; GR, Grimsby; FH, Farmington Hills; SA, Sakai; HT, Hunter; YE, Yerseke; DH, Den Haag; OS, Osaka; AP, Apeldoorn; NO, New Orleans; SY, Sydney.

As antigenic site A is the most variable site and plays a differential role in the antigenic diversification of GII.4 variants (5, 6, 9, 11), we sought to determine what residues play a major role in the binding from three mAbs (NORO-263, -315B, and 250B) mapping to this site. Due to the extensive cross-reactivity of NORO-123, we were unable to further map this antibody. Thus, we divided the antigenic site into three regions or motifs: designated A(I), A(II), or A(III), consisting of residues 294 and 295, 296-298 and 368, or 372 and 373, respectively (**Figure 4A**). Reactivity of the mAbs against Sydney 2012 mutants for the residues forming those three motifs showed that A(I) and A(II) were important for the binding of NORO-263 and -315B, with the loss of binding against 2012:A(I)^02^ and 2012:A(II)^02^ VLPs, while A(II) was the sole binding site of NORO-250B (**Figure 4B**). To determine the residues in antigenic site C that are important for NORO-115 binding, we tested reactivity against two FH 2002 mutants: FH 2002: E376Q and FH 2002: G340A/E376Q (**Figure 4C**) (11). NORO-115 showed reduced binding against the mutant VLPs, with a slightly greater loss of binding with the double mutant (**Figure 4D**). Thus, although residue 376 plays a major role in the NORO-115 binding site, other residues, such as 340, should not be ruled out. The sequence alignment of antigenic site C suggests a role for residues 340 and 341 (**Figure 4E**, **S5 Figure**). Variability on site 340 explains the lack of reactivity of this mAb against Hunter 2004 VLPs and the decrease in reactivity against Yerseke 2006a VLPs, while an asparagine on residue 341 may hinder the binding to New Orleans 2009 VLPs. Indeed, binding of NORO-115 is restored with a New Orleans 2009 virus, Ehime2/2009 (15), that does not present the N341 mutation (**S6 Figure**). We also examined the antigenic site A residues to further elucidate important residues for antibodies targeting this region (**Figure 4E**; **S5 Figure**). Each motif of antigenic site A presented at least one highly variable residue: 294 [A(I)], 298 and 368 [A(II)], or 372 [A(III)]. Each motif showed ≥ 4 mutations, explaining the different binding profiles observed for NORO-263, -315B, and -250B (**Figure 2**). Together, the reactivity patterns of mAbs against multiple variants reveal a complex relationship between antibody binding and the mutational profile of the residues forming the antigenic sites.

## Discussion

The humoral immune response, in particular the production of blocking and neutralizing antibodies, is thought to play a major role in both norovirus clearance and protection (7, 37, 38). Thus, a better understanding of the mechanisms governing host antibody responses to fight the diversity presented by norovirus is necessary to inform the development of therapeutics and the design of new vaccine candidates. In this study, we characterized a panel of human mAbs that were isolated from individuals naturally infected with a GII.4 Sydney virus in 2013. All these mAbs are potentially protective, as they show strong carbohydrate blocking activity against multiple GII.4 variants and neutralizing activity as measured by the human enteroid *in vitro* culture system for noroviruses. Notably, the mAbs neutralized a GII.4 Sydney virus that was detected five years after predominance of this variant and that contains three amino acid mutations in the major capsid protein, as compared to the prototype Sydney 2012 virus. Although changes could result in antigenic differences, intra-variant changes seem to have a minimal effect on the antigenic characteristics of GII.4 viruses (15).

All six mAbs were specific to GII.4 viruses and exhibited carbohydrate blockade against multiple GII.4 viruses representing the major and minor pandemic variants that have emerged since the mid-1980’s. Despite mapping to the highly variable antigenic site A, mAb NORO-123 bound to all GII.4 variants tested, including Camberwell 1987. This mAb also exhibited strong carbohydrate blocking activity against all variants. Interestingly, while the variants tested showed up to 7 amino acid differences on this antigenic site (**Figure 4**), it took mutations in all 8 residues (12ΔA VLPs) to abolish binding from this antibody, showing that this antibody can accommodate multiple mutations at this site. Structural analyses will confirm whether NORO-123 binds directly to or indirectly interacts with antigenic site A. Regardless, these properties make NORO-123 a good candidate for a potential therapeutic molecule, and further research is warranted for this mAb.

The extensive cross-reactivity of the mAbs described here contrasts with our prior data that suggested little-to-no cross-blockade of contemporary versus historical variants that emerged prior to 2006 (15). However, the development of cross-reactive antibodies might be influenced by the nature (*e.g*., immunization versus infection) and the frequency of exposure to the antigen. Thus, the mAbs described here were developed from adult individuals who likely had a history of previous norovirus infections that guided their immune response towards the production of cross-reactive antibodies.

One major difference between the cross-reactive antibodies described here and those previously described is their binding site on the VP1 protein. While A1431 and NORO-320 bind to a highly conserved region at the P1/P2 subdomain interface (25, 26), the mAbs described in this study mapped to antigenic sites A (NORO-123, -263, -315B, -250B) and C (NORO-115). These antigenic sites are highly variable, and mutational patterns on these sites are correlated with the emergence of new GII.4 variants (5, 9, 39). In addition, antigenic site A is near the HBGA binding site (7, 10, 40), suggesting that antibodies that bind this region prevent the binding of the viral particle to the HBGA attachment factors. Using sequence analyses and a panel of mutant VLPs with swapped residues within antigenic site A, we further narrowed down the residues involved in the binding of NORO-263, -315B, and -250B. Interestingly, all three antibodies had differing binding patterns within antigenic site A. We have shown previously that murine mAbs that bind to A(I) or A(II) potently neutralize a GII.4 Sydney virus, while a murine mAb that binds to A(III) only neutralized at a high concentration (13), suggesting that motifs A(I) and A(II) are major targets of neutralizing antibodies. Our data supports the finding that certain “anchor” residues within antigenic site A are important for the binding of neutralizing antibodies (25, 41). A previous study detected a human mAb that mapped to antigenic site D and showed partial cross-reactivity against contemporary GII.4 variants (Yerseke 2006a, DenHaag 2006b, and New Orleans 2009) and the Hunter 2004 variant, showing that multiple variable antigenic sites could elicit cross-reactive antibodies (6). Other studies have shown limited cross-reactivity for human mAbs mapping to sites A and/or G, albeit with distinct clustering in reactivity against either archival or contemporary variants (6, 25, 42). One mouse mAb, GII.4-2006-G3, which mapped to antigenic site A, presented blocking activity against the FH 2002, DH 2006, and NO 2009 variants, but was unable to block variants prior to 2002 and the pandemic SY 2012 variant (41, 43). To our knowledge, this is the first study to show highly cross-reactive human antibodies to archival and contemporary variants that map to antigenic sites A or C. These results suggest that the immune response could be tailored to areas of VP1 that, despite presenting some diversification, would elicit cross-protective antibodies.

Taken together, these data suggest that human antibodies that bind to highly variable antigenic sites on the norovirus capsid are still able to recognize GII.4 variants that emerged decades prior or that present extensive diversification. These antibodies present neutralizing and potent HBGA blocking activity against contemporary and historical GII.4 variants, offering valuable insights on the cross-reactivity of the humoral immune response following natural infection. Studies on the role of prior infections on antibody responses would inform vaccine design to mitigate GII.4 infection, the most common norovirus genotype infecting humans.

## Data availability statement

Antibodies described in this paper are available for distribution for nonprofit use using templated documents from Association of University Technology Managers “Toolkit MTAs”, available at: https://autm.net/surveys-and-tools/agreements/material-transfer-agreements/mta-toolkit. Further information and requests for antibodies should be directed to and will be fulfilled by James E. Crowe, Jr. (james.crowe@vumc.org). VLPs described in this paper are available upon request to Gabriel I. Parra (gabriel.parra@fda.hhs.gov).

## Author contributions

LF-S, KT, JC, and GP contributed to conception and design of the study. LF-S, KT, and KP performed study-related experiments. GA and JC provided monoclonal antibodies used in the study. LF-S, KT, JK, and GP produced reagents and materials used in the study. LF-S wrote the first draft of the manuscript. KT and GP wrote sections of the manuscript. All authors contributed to manuscript revision and approved the submitted version.

## Funding

Financial support for this work was provided by the Food and Drug Administration intramural funds (program number Z01 BK 04012 LHV to GP). This work was also supported by a pilot and feasibility grant from the Vanderbilt University Medical Center’s Digestive Disease Research Center supported by NIH grants P30 DK058404. The project described was supported by The Vanderbilt Institute for Clinical and Translational Research (VICTR) is funded by the National Center for Advancing Translational Sciences (NCATS) Clinical Translational Science Award (CTSA) Program, Award Number 5UL1TR002243-03. The content is solely the responsibility of the authors and does not necessarily represent the official views of the NIH. GA was supported through the Vanderbilt Trans-Institutional Program (TIP) “Integrating Structural Biology with Big Data for next Generation Vaccines” and NIH grant F31 Al129357.

## Acknowledgments

We thank Dr. Yamei Gao (DVP, CBER, FDA) for confirming the integrity of the VLPs used throughout this study *via* transmission electron microscopy. We also thank Nurgun Kose and Robin Bombardi in the Crowe laboratory at Vanderbilt for excellent technical support.

## Conflict of interest

JC has served as a consultant for Luna Labs USA, Merck Sharp & Dohme Corporation, Emergent Biosolutions, and GlaxoSmithKline, and is a member of the Scientific Advisory Board of Meissa Vaccines, a former member of the Scientific Advisory Board of Gigagen Grifols and is founder of IDBiologics. The laboratory of JC received unrelated sponsored research agreements from AstraZeneca, Takeda, and IDBiologics during the conduct of the study. Vanderbilt University has applied for patents for some of the antibodies in this paper.

The remaining authors declare that the research was conducted in the absence of any commercial or financial relationships that could be construed as a potential conflict of interest.

## Publisher’s note

All claims expressed in this article are solely those of the authors and do not necessarily represent those of their affiliated organizations, or those of the publisher, the editors and the reviewers. Any product that may be evaluated in this article, or claim that may be made by its manufacturer, is not guaranteed or endorsed by the publisher.

## References

[1] Green KY. Norovirus infection in immunocompromised hosts. Clin Microbiol Infect (2014) 20(8):717–23. doi: 10.1111/1469-0691.12761 PMC11036326 25040790

[2] Chhabra P de Graaf M Parra GI Chan MC Green K Martella V. Updated classification of norovirus genogroups and genotypes. J Gen Virol (2019) 100(10):1393–406. doi: 10.1099/jgv.0.001318 PMC7011714 31483239

[3] Parra GI Squires RB Karangwa CK Johnson JA Lepore CJ Sosnovtsev SV. Static and evolving norovirus genotypes: Implications for epidemiology and immunity. PloS Pathog (2017) 13(1):e1006136. doi: 10.1371/journal.ppat.1006136 28103318 PMC5283768

[4] Parra GI. Emergence of norovirus strains: A tale of two genes. Virus Evol (2019) 5(2):vez048. doi: 10.1093/ve/vez048 32161666 PMC6875644

[5] Tohma K Lepore CJ Gao Y Ford-Siltz LA Parra GI. Population genomics of GII.4 noroviruses reveal complex diversification and new antigenic sites involved in the emergence of pandemic strains. MBio (2019) 10(5):e02202-19. doi: 10.1128/mBio.02202-19 31551337 PMC6759766

[6] Lindesmith LC Beltramello M Donaldson EF Corti D Swanstrom J Debbink K. Immunogenetic mechanisms driving norovirus GII.4 antigenic variation. PloS Pathog (2012) 8(5):e1002705. doi: 10.1371/journal.ppat.1002705 22615565 PMC3355092

[7] Ford-Siltz LA Tohma K Parra GI. Understanding the relationship between norovirus diversity and immunity. Gut Microbes (2021) 13(1):1–13. doi: 10.1080/19490976.2021.1900994 PMC8018473 33783322

[8] Koromyslova AD Morozov VA Hefele L Hansman GS. Human norovirus neutralized by a monoclonal antibody targeting the histo-blood group antigen pocket. J Virol (2019) 93(5):e02174-18. doi: 10.1128/JVI.02174-18 30541855 PMC6384083

[9] Allen DJ Gray JJ Gallimore CI Xerry J Iturriza-Gomara M. Analysis of amino acid variation in the P2 domain of the GII-4 norovirus VP1 protein reveals putative variant-specific epitopes. PloS One (2008) 3(1):e1485. doi: 10.1371/journal.pone.0001485 18213393 PMC2194622

[10] Debbink K Donaldson EF Lindesmith LC Baric RS. Genetic mapping of a highly variable norovirus GII.4 blockade epitope: potential role in escape from human herd immunity. J Virol (2012) 86(2):1214–26. doi: 10.1128/JVI.06189-11 PMC3255819 22090110

[11] Parra GI Abente EJ Sandoval-Jaime C Sosnovtsev SV Bok K Green KY. Multiple antigenic sites are involved in blocking the interaction of GII.4 norovirus capsid with ABH histo-blood group antigens. J Virol (2012) 86(13):7414–26. doi: 10.1128/JVI.06729-11 PMC3416322 22532688

[12] Lindesmith LC Debbink K Swanstrom J Vinje J Costantini V Baric RS. Monoclonal antibody-based antigenic mapping of norovirus GII.4-2002. J Virol (2012) 86(2):873–83. doi: 10.1128/JVI.06200-11 PMC3255811 22090098

[13] Tohma K Ford-Siltz LA Kendra JA Parra GI. Dynamic immunodominance hierarchy of neutralizing antibody responses to evolving GII.4 noroviruses. Cell Rep (2022) 39(2):110689. doi: 10.1016/j.celrep.2022.110689 35417705

[14] Shanker S Czako R Sankaran B Atmar RL Estes MK Prasad BV. Structural analysis of determinants of histo-blood group antigen binding specificity in genogroup I noroviruses. J Virol (2014) 88(11):6168–80. doi: 10.1128/JVI.00201-14 PMC4093872 24648450

[15] Kendra JA Tohma K Ford-Siltz LA Lepore CJ Parra GI. Antigenic cartography reveals complexities of genetic determinants that lead to antigenic differences among pandemic GII.4 noroviruses. Proc Natl Acad Sci U S A (2021) 118(11):e2015874118. doi: 10.1073/pnas.2015874118 33836574 PMC7980451

[16] Prasad BV Hardy ME Dokland T Bella J Rossmann MG Estes MK. X-Ray crystallographic structure of the Norwalk virus capsid. Science (1999) 286(5438):287–90. doi: 10.1126/science.286.5438.287 10514371

[17] Rockx BH Vennema H Hoebe CJ Duizer E Koopmans MP. Association of histo-blood group antigens and susceptibility to norovirus infections. J Infect Dis (2005) 191(5):749–54. doi: 10.1086/427779 15688291

[18] Le Pendu J Ruvoen-Clouet N. Fondness for sugars of enteric viruses confronts them with human glycans genetic diversity. Hum Genet (2020) 139(6-7):903–10. doi: 10.1007/s00439-019-02090-w 31760489

[19] Haga K Ettayebi K Tenge VR Karandikar UC Lewis MA Lin SC. Genetic manipulation of human intestinal enteroids demonstrates the necessity of a functional fucosyltransferase 2 gene for secretor-dependent human norovirus infection. mBio (2020) 11(2):e00251-20. doi: 10.1128/mBio.00251-20 32184242 PMC7078471

[20] Bok K Parra GI Mitra T Abente E Shaver CK Boon D. Chimpanzees as an animal model for human norovirus infection and vaccine development. Proc Natl Acad Sci U S A (2011) 108(1):325–30. doi: 10.1073/pnas.1014577107 PMC3017165 21173246

[21] Reeck A Kavanagh O Estes MK Opekun AR Gilger MA Graham DY. Serological correlate of protection against norovirus-induced gastroenteritis. J Infect Dis (2010) 202(8):1212–8. doi: 10.1086/656364 PMC2945238 20815703

[22] Atmar RL Bernstein DI Harro CD Al-Ibrahim MS Chen WH Ferreira J. Norovirus vaccine against experimental human Norwalk virus illness. N Engl J Med (2011) 365(23):2178–87. doi: 10.1056/NEJMoa1101245 PMC3761795 22150036

[23] Ford-Siltz LA Wales S Tohma K Gao Y Parra GI. Genotype-specific neutralization of norovirus is mediated by antibodies against the protruding domain of the major capsid protein. J Infect Dis (2020) 225 (7):1205–14. doi: 10.1093/infdis/jiaa116 32179892

[24] van Loben Sels JM Green KY. The antigenic topology of norovirus as defined by b and T cell epitope mapping: Implications for universal vaccines and therapeutics. Viruses (2019) 11(5):432. doi: 10.3390/v11050432 PMC6563215 31083353

[25] Lindesmith LC McDaniel JR Changela A Verardi R Kerr SA Costantini V. Sera antibody repertoire analyses reveal mechanisms of broad and pandemic strain neutralizing responses after human norovirus vaccination. Immunity (2019) 50(6):1530–41 e8. doi: 10.1016/j.immuni.2019.05.007 31216462 PMC6591005

[26] Alvarado G Salmen W Ettayebi K Hu L Sankaran B Estes MK. Broadly cross-reactive human antibodies that inhibit genogroup I and II noroviruses. Nat Commun (2021) 12(1):4320. doi: 10.1038/s41467-021-24649-w 34262046 PMC8280134

[27] Alvarado G Ettayebi K Atmar RL Bombardi RG Kose N Estes MK. Human monoclonal antibodies that neutralize pandemic GII. 4 Noroviruses Gastroenterol (2018) 155(6):1898–907. doi: 10.1053/j.gastro.2018.08.039 PMC6402321 30170116

[28] Tamura K Dudley J Nei M Kumar S. MEGA4: Molecular evolutionary genetics analysis (MEGA) software version 4. 0 Mol Biol Evol (2007) 24(8):1596–9. doi: 10.1093/molbev/msm092 17488738

[29] Pettersen EF Goddard TD Huang CC Couch GS Greenblatt DM Meng EC. UCSF chimera–a visualization system for exploratory research and analysis. J Comput Chem (2004) 25(13):1605–12. doi: 10.1002/jcc.20084 15264254

[30] Yu C Wales SQ Mammel MK Hida K Kulka M. Optimizing a custom tiling microarray for low input detection and identification of unamplified virus targets. J Virol Methods (2016) 234:54–64. doi: 10.1016/j.jviromet.2016.03.013 27033182

[31] Atmar RL Ettayebi K Ayyar BV Neill FH Braun RP Ramani S. Comparison of microneutralization and histo-blood group antigen-blocking assays for functional norovirus antibody detection. J Infect Dis (2020) 221(5):739–43. doi: 10.1093/infdis/jiz526 PMC8483564 31613328

[32] Karangwa CK Parra GI Bok K Johnson JA Levenson EA Green KY. Sequential gastroenteritis outbreaks in a single year caused by norovirus genotypes GII.2 and GII.6 in an institutional setting. Open Forum Infect Dis (2017) 4(4):ofx236. doi: 10.1093/ofid/ofx236 30349844 PMC5903414

[33] Parra GI Green KY. Sequential gastroenteritis episodes caused by 2 norovirus genotypes. Emerg Infect Dis (2014) 20(6):1016–8. doi: 10.3201/eid2006.131627 PMC4036768 24857806

[34] Sakon N Yamazaki K Nakata K Kanbayashi D Yoda T Mantani M. Impact of genotype-specific herd immunity on the circulatory dynamism of norovirus: a 10-year longitudinal study of viral acute gastroenteritis. J Infect Dis (2015) 211(6):879–88. doi: 10.1093/infdis/jiu496 25210139

[35] Saito M Goel-Apaza S Espetia S Velasquez D Cabrera L Loli S. Multiple norovirus infections in a birth cohort in a Peruvian periurban community. Clin Infect Dis (2014) 58(4):483–91. doi: 10.1093/cid/cit763 PMC3905757 24300042

[36] Chhabra P Rouhani S Browne H Yori PP Salas MS Olortegui MP. Homotypic and heterotypic protection and risk of re-infection following natural norovirus infection in a highly endemic setting. Clin Infect Dis (2021) 72(2):222–9. doi: 10.1093/cid/ciaa019 PMC7840104 33501947

[37] Atmar RL Ramani S Estes MK. Human noroviruses: recent advances in a 50-year history. Curr Opin Infect Dis (2018) 31(5):422–32. doi: 10.1097/QCO.0000000000000476 30102614

[38] Ramani S Estes MK Atmar RL. Correlates of protection against norovirus infection and disease-where are we now, where do we go? PloS Pathog (2016) 12(4):e1005334. doi: 10.1371/journal.ppat.1005334 27115358 PMC4846004

[39] Lindesmith LC Donaldson EF Lobue AD Cannon JL Zheng DP Vinje J. Mechanisms of GII.4 norovirus persistence in human populations. PloS Med (2008) 5(2):e31. doi: 10.1371/journal.pmed.0050031 18271619 PMC2235898

[40] Tan M Huang P Xia M Fang PA Zhong W McNeal M. Norovirus p particle, a novel platform for vaccine development and antibody production. J Virol (2011) 85(2):753–64. doi: 10.1128/JVI.01835-10 PMC3020015 21068235

[41] Lindesmith LC Costantini V Swanstrom J Debbink K Donaldson EF Vinje J. Emergence of a norovirus GII.4 strain correlates with changes in evolving blockade epitopes. J Virol (2013) 87(5):2803–13. doi: 10.1128/JVI.03106-12 PMC3571402 23269783

[42] Mallory ML Lindesmith LC Graham RL Baric RS. GII.4 human norovirus: Surveying the antigenic landscape. Viruses (2019) 11(2)177. doi: 10.3390/v11020177 PMC6410000 30791623

[43] Lindesmith LC Donaldson EF Baric RS. Norovirus GII.4 strain antigenic variation. J Virol (2011) 85(1):231–42. doi: 10.1128/JVI.01364-10 PMC3014165 20980508

