# Supplementary figures and images for: Cross-reactive neutralizing human monoclonal antibodies mapping to variable antigenic sites on the norovirus major capsid protein
Source: Front Immunol. 2022 Oct 25;13:1040836. doi: 10.3389/fimmu.2022.1040836 (PMC9641292; doi:10.3389/fimmu.2022.1040836)

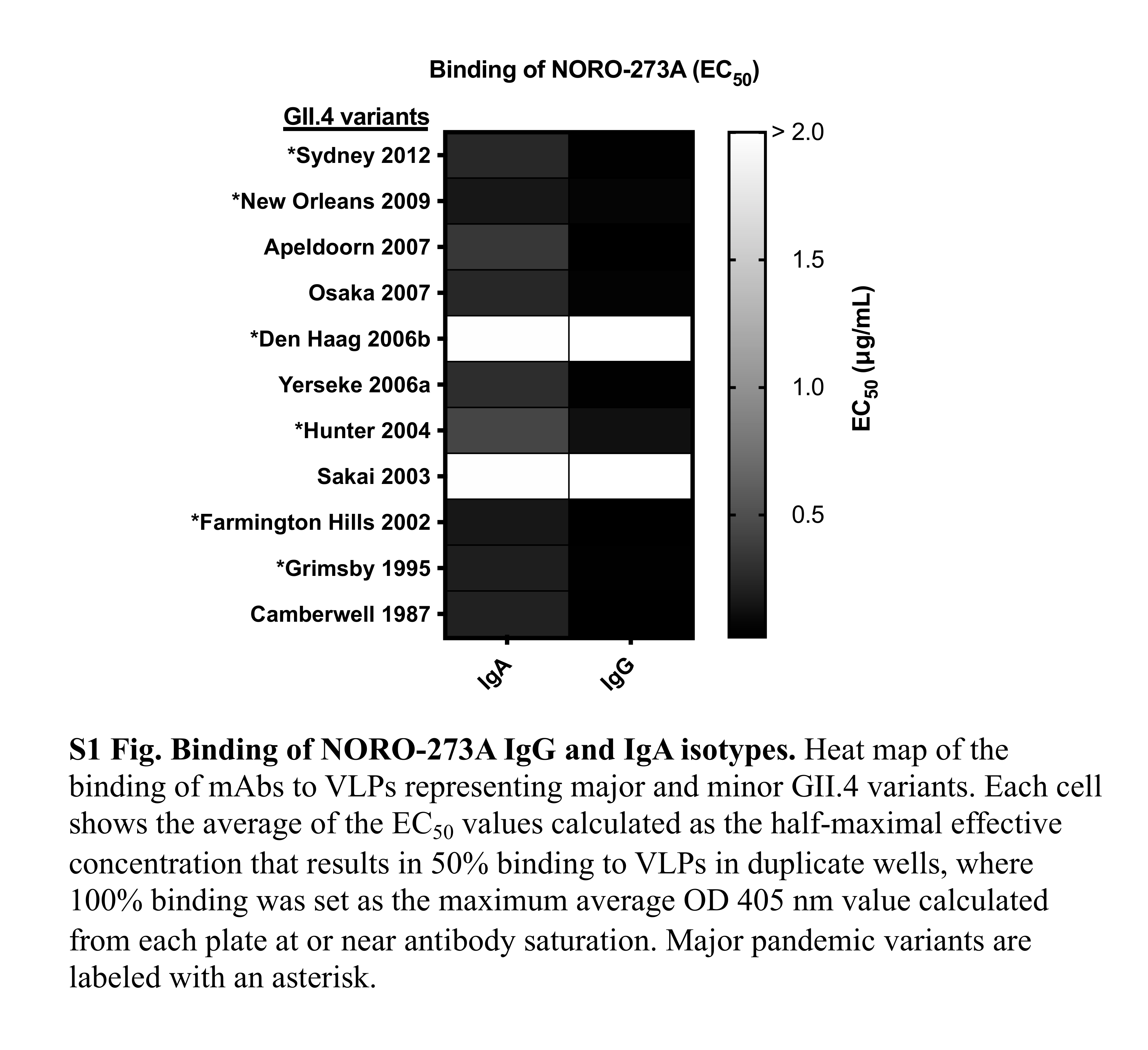

Supplement: Supplementary file 1 [file Image_1.tif]

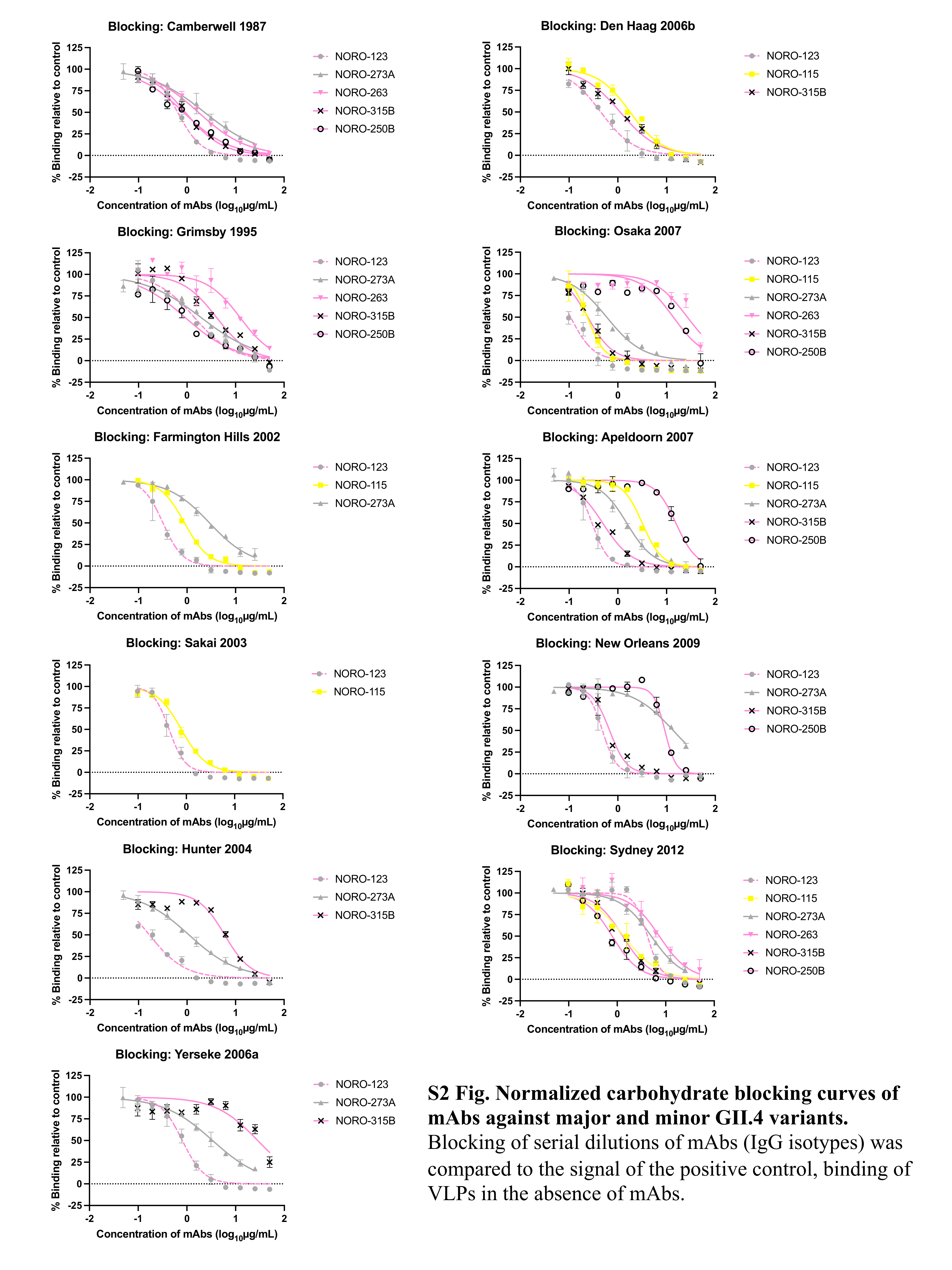

Supplement: Supplementary file 2 [file Image_2.tif]

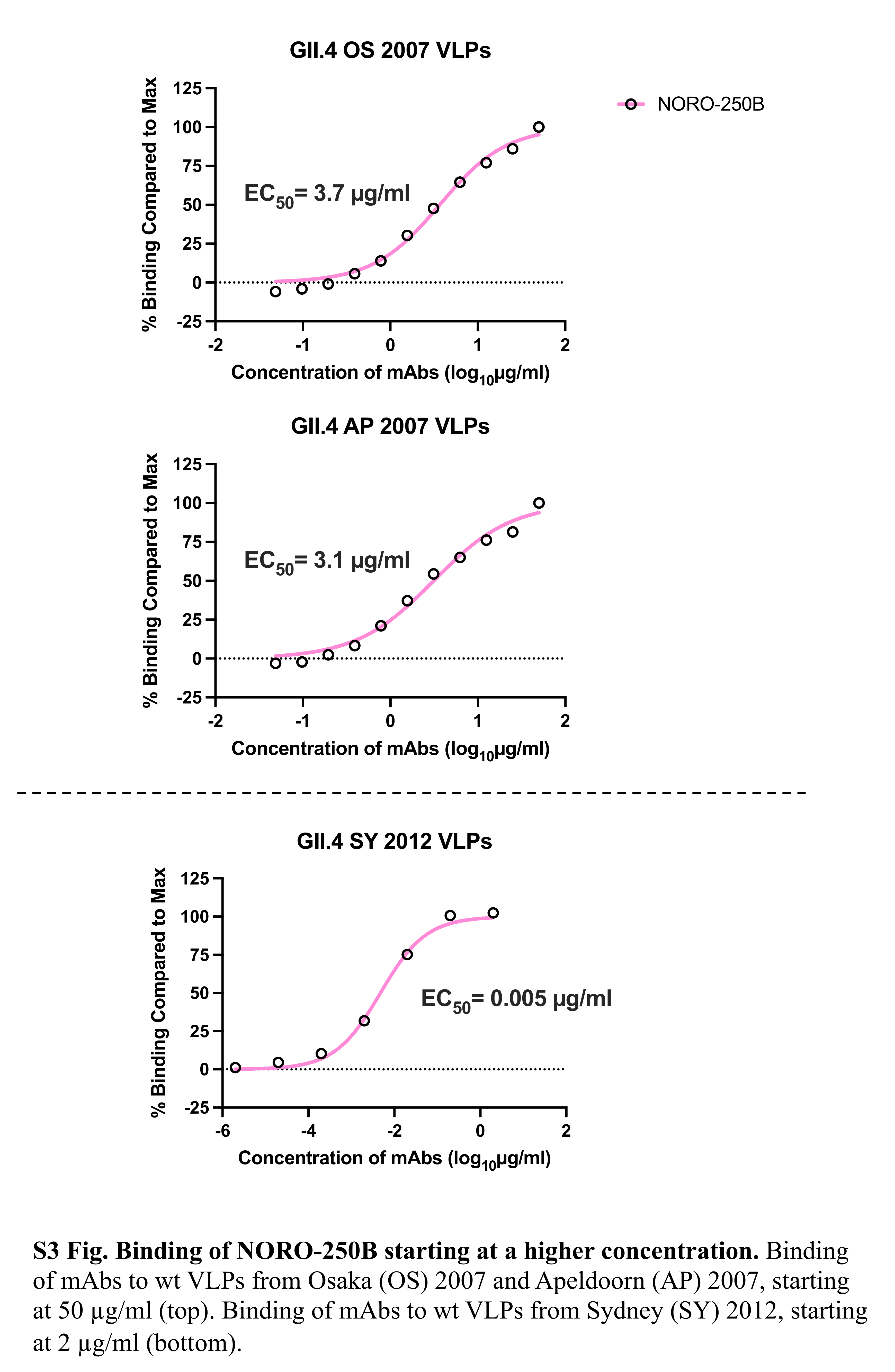

Supplement: Supplementary file 3 [file Image_3.tif]

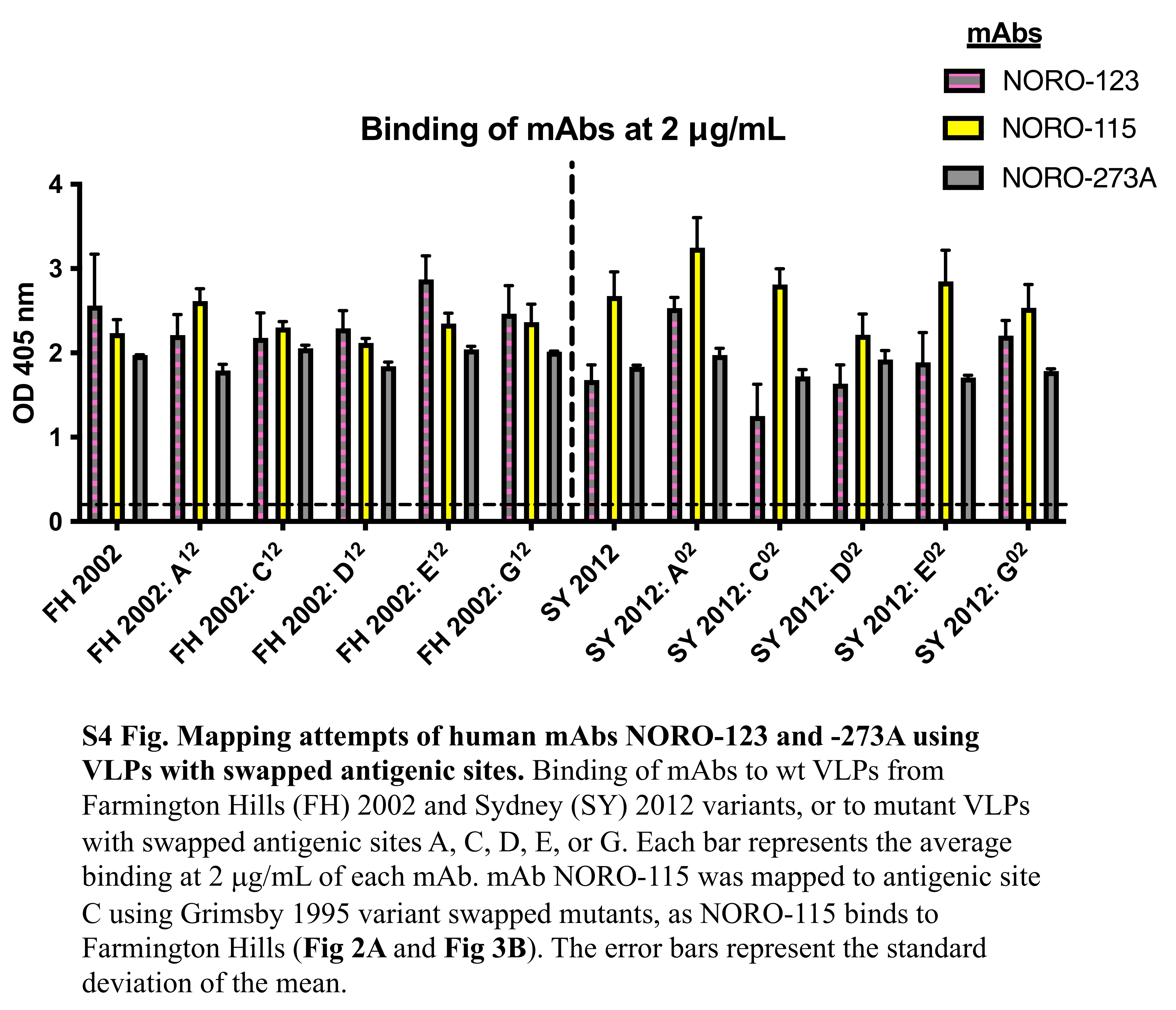

Supplement: Supplementary file 4 [file Image_4.tif]

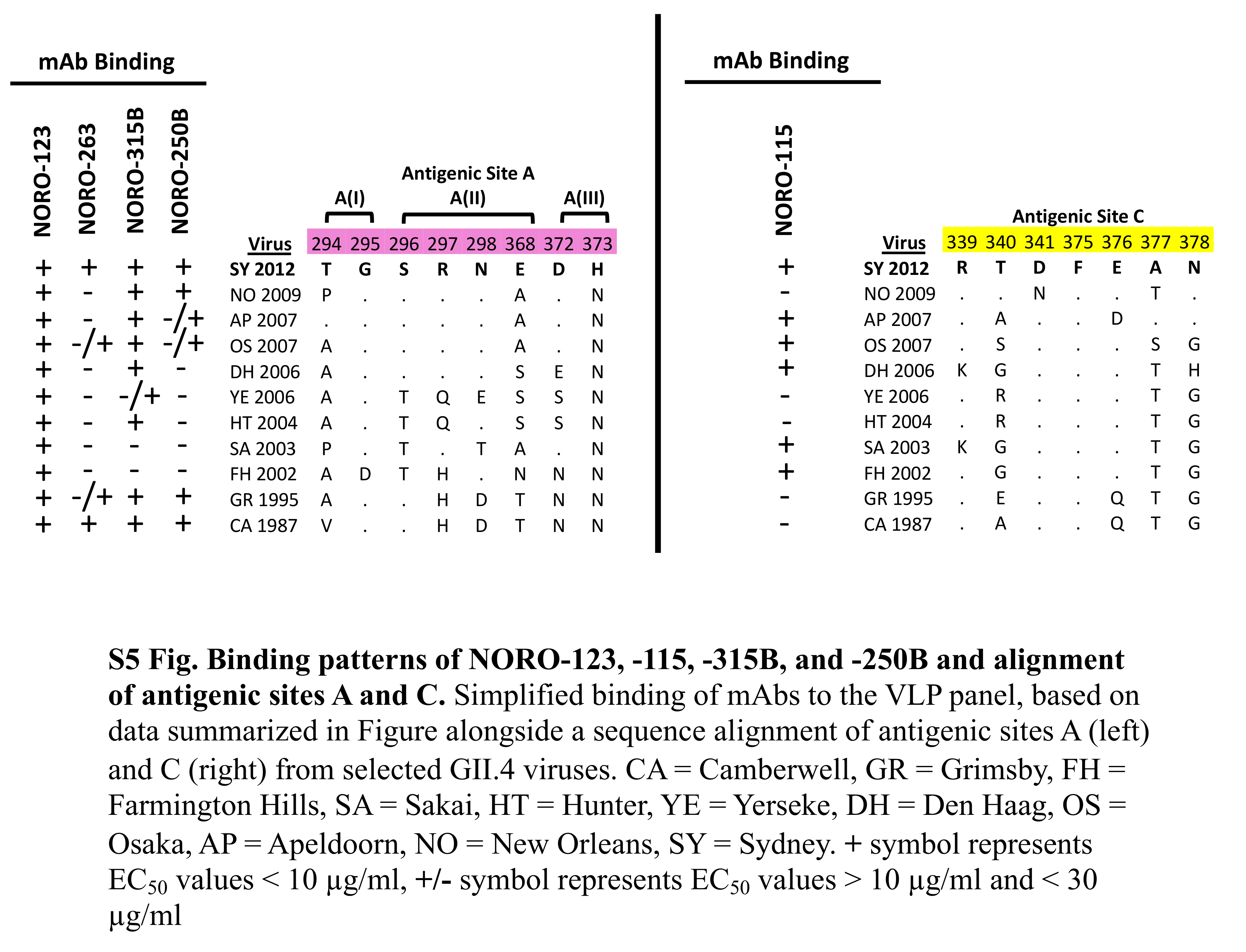

Supplement: Supplementary file 5 [file Image_5.tif]

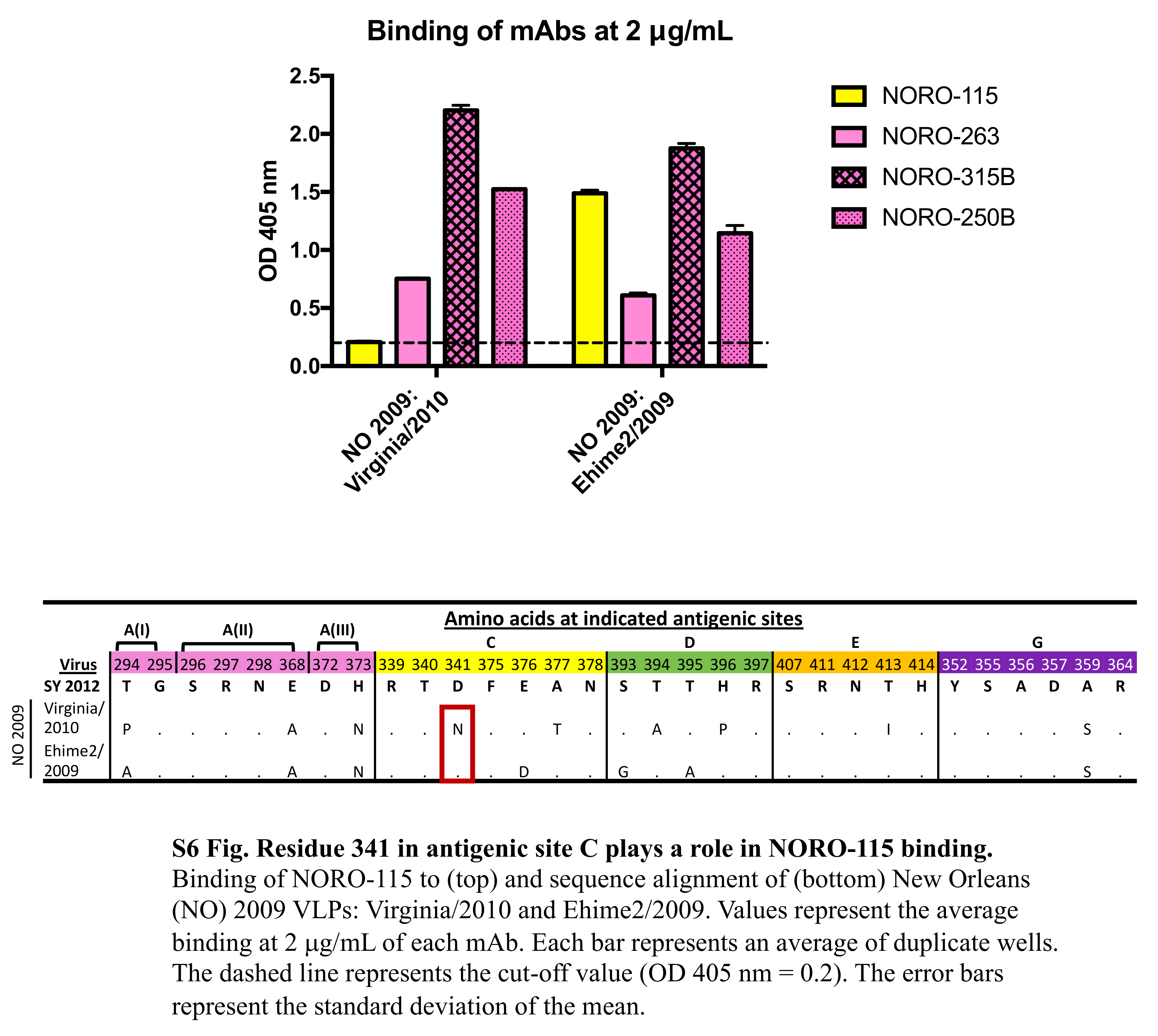

Supplement: Supplementary file 6 [file Image_6.tif]
